# Ceramic-on-metal coupling in THA: long term clinical and radiographic outcomes using two different short stems

**DOI:** 10.1186/s12891-022-05077-3

**Published:** 2022-02-22

**Authors:** Giandomenico Logroscino, Michela Saracco, Giulio Maccauro, Andrea Urbani, Domenico Ciavardelli, Ada Consalvo, Daniele Ferraro, Francesco Falez

**Affiliations:** 1 Mininvasive and Computer-Assisted Orthopaedic Surgery, Department of Life, Health and Environmental Sciences, University of L’Aquila, L’Aquila, Italy; 2 Department of Orthopaedics, Catholic University of Rome-Fondazione Policlinico Universitario A. Gemelli IRCCS, Largo F. Vito,1, 00168 Rome, Italy; 3 Institute of Biochemistry and Clinical Biochemistry, Catholic University of Rome-Fondazione Policlinico Universitario A. Gemelli IRCCS, Rome, Italy; 4 School of Medicine and Surgery, University “Kore” of Enna, Enna, Italy; 5 Center for Advanced Studies and Technology (C.A.S.T.), University “G. d’Annunzio” of Chieti-Pescara, Chieti, Italy; 6 Department of Orthopaedics and Traumatology, ASL Roma 1, S. Filippo Neri Hospital and S. Spirito Hospital, Rome, Italy

**Keywords:** Total hip arthroplasty, Hard-on-hard bearing, Ceramic-on-metal, Short femoral stem

## Abstract

**Background:**

Hip prosthetic replacement surgery is the gold standard for patients affected by symptomatic osteoarthritis. The ceramic-on-metal hybrid hard-on-hard bearing was initially launched on the market with the purpose of reducing adhesive and corrosion wear, loss of metal debris and ions and risk of fracture and squeaking. However, this bearing was withdrawn from the market, in the apprehension of local and systemic toxicity. The aim of this study is to evaluate the reliability and safety of ceramic-on-metal bearing at long term follow-up.

**Methods:**

From 2 cohorts of patients suffering of hip osteoarthritis who underwent total hip arthroplasty using ceramic-on-metal bearing with two different short stems, 19 of the GROUP A and 25 of the GROUP B were suitable for this study. All patients were compared clinically using the Harris Hip Score (HHS), Western Ontario and McMaster Universities Osteoarthritis Index (WOMAC), visual analogue scale (VAS), 12-item Short Form Health Survey (SF12P/M), and radiographically. Blood samples were collected in order to evaluate chromium and cobalt ions level. The two groups were compared in terms of metal ions blood levels, and finally all the implanted prostheses were compared with a healthy control group.

**Results:**

All the implanted stems were well-positioned and osseointegrated at a mean follow-up of 114 months. Improvements were observed for all clinical scores comparing preoperative and postoperative values in both groups. Radiographic evaluation showed a good ability to restore proper articular geometry. Chromium and cobalt ion analysis revealed values below the safety threshold except for 1 case in GROUP A (cup malposition) and 2 cases in GROUP B (6.1%). No revision occurred.

**Conclusions:**

Ceramic-on-metal bearing is safe and reliable at long term follow-up in association to short stems arthroplasty, if the implant is correctly positioned. Chromium and cobalt metal ions blood levels evaluation should be performed annually.

## Background

Symptomatic hip osteoarthritis severely compromises the quality of life of an important slice of the population. Prosthetic replacement surgery is nowadays the gold standard for those patients in whom conservative treatment have lost its effectiveness. This technique, introduced in the seventies of the twentieth century, guarantees high standards of safety and reliability. This was possible thanks to the technologic improvement of the materials. Different bearing options were developed to ensure low wear, high biological safety of debris and stability of the hip prosthetic implants. Ceramic-on-Ceramic (CoC) coupling is certainly the most reliable and long-lasting one, thanks to the development of Alumina and Zirconia-based composites [1].

This bearing is currently the only hard-on-hard coupling on the market widely used with negligible wear rates [2–4]. Indeed, Ceramic-on-Metal (CoM) and Metal-on-Metal (MoM) bearings have been dismissed due to the risk of release of chromium (Cr) and cobalt (Co) ions and consequent local and systemic toxicity, especially in the event of implant mal-positioning.

While CoC and MoM bearings are desirable due to markedly decreased wear rates, the first one is associated with rare risk of head or liner fracture (1,3/ 100.000) [5] and audible noise generation (squeaking), in most cases a transient condition [6], and the latter with increased risk of adverse local tissue reactions (ALTR) due to metal debris, which was histologically described as aseptic lymphocytic vasculitis-associated lesion, tissue necrosis and pseudotumor [7, 8].

If this is certainly true for MoM, the same cannot be said for the hard-on-hard hybrid CoM bearing, a ceramic femoral head articulating with a metal acetabular liner. In fact, CoM was previously withdrawn from the market, in the idea that the ceramic could wear out the metal liner and favour the release of metal ions. This hybrid hard-on-hard bearing was initially launched on the market with the purpose of reducing adhesive and corrosion wear, loss of metal debris and ions because only one half of the implant is made of metal. In addition, CoM has a reduced risk of fracture and squeaking, compared to CoC [9]. What is particularly interesting, however, is that CoM has been withdrawn without scientific evidences having demonstrated the increased risk of complications. Indeed, CoM has shown excellent clinical and radiographic outcomes: serum Cr-Co ions level was found to be mildly above baseline, but always inferior to the Medicines and Healthcare Products Regulatory Agency (MHRA) threshold of 7 μg / l [10]. Little literature already exists regarding the real risk represented by the implantation of this bearing, and indeed, the few available studies seem to support reliability and safety. Information on metal ion values following CoM implantation is limited due to the fact that there is a focus on MoM devices regarding metal wear and systemic exposure with unknown long-term effects. Many reports showed good medium-term clinical and radiographic results of CoM, with metal ions elevation within safety and acceptable limits [11, 12] but none of them report results at long term follow-up.

The aim of this study is to further evaluate at long term follow-up the reliability and safety, intended as a low risk of incurring into toxicity from Cr and Co, of CoM bearing by comparing clinical and radiographic results and serum Cr-Co ions level of two cohorts of patients who had previously undergone THA with two different short femoral stems and CoM bearing. The two stems analyzed are both short, but with different characteristics. The Metha stem (Aesculap implant systems - B. Braun company) differ from the Proxima stem (De Puy international, Leeds, UK) because it is a neck-retaining one, requiring a more complex implant technique and high femoral osteotomy. Therefore, we wanted to evaluate how possible procedural difficulties, positioning errors of the stem or cup could affect the risk of release of Cr-Co metal ions. The value of the serum ions of the two groups have been compared with each other and with the values obtained from a healthy control group, homogeneous for sex, age and exposure history [13].

## Methods

On a basis of 124 CoM patients, we analysed 2 cohorts. The first one included 37 cases who underwent to surgery between 2007 and 2013 with the same implant: Proxima, an uncemented anatomic proximally fixed short monoblock femoral stem, and Pinnacle acetabular shell with Ultramet Co-Cr-Mo alloy liner (De Puy International, Leeds, UK) and 36-mm femoral head made of zirconia-toughened alumina ceramic (Biolox Delta, Ceramtec, Plochingen, Germany). The second one included 87 cases who received between 2008 and 2010 Metha (Aesculap Implant Systems – B. Braun company), an uncemented neck-retaining, monoblock stem, and Pinnacle acetabular shell with Ultramet Co-Cr-Mo alloy liner (De Puy International, Leeds, UK) and 36-mm femoral head made from zirconia-toughened alumina ceramic (Biolox Delta, Ceramtec, Plochingen, Germany).

Non-inflammatory arthritis, primary osteoarthritis (OA), post-traumatic osteoarthritis and avascular necrosis (AVN) and mild developmental dysplasia of the hip (DDH) were the main indication for surgery. Exclusion criteria were metal allergy, acute or chronic kidney failure, other metallic implants, inflammatory arthritis (rheumatoid arthritis), infections, previous hip revision surgery, neurological deficits affecting movement, dementia, occupational or dietary Cr or Co exposure.

Finally, we retrospectively enrolled in the study 44 patients. Of the others, the majority was lost due to patient decision not to attend, frailty, death or familiar unavailability to collaborate. Of these, when possible, telephone informations were collected regarding the hip status of health. Of the enrolled patients, the first cohort, Proxima femoral stem implanted, (GROUP A) was a total of 19 patients, 10 male and 9 females. 18 patients were affected by primary hip osteoarthritis and 1 by osteoarthritis secondary to mild DDH. 25 patients were enrolled from the second cohort: Metha femoral stem implanted, (GROUP B), 17 males and 8 females. 17 patients were affected by primary hip osteoarthritis and 8 by hip osteoarthritis secondary to mild DDH, proximal femoral and acetabular fractures and epiphysiolysis.

The same surgeon performed all the arthroplasties of the GROUP A via a posterior-lateral approach. Surgical procedures of the GROUP B were performed by another senior surgeon using the direct lateral approach. Low-molecular-weight heparin was used as an anticoagulant during the first 5 weeks after surgery, starting from 6 h after the procedure. 2 g of cefazolin was administered during anaesthesia induction and tranexamic acid was used for bleeding control.

8 patients of the GROUP A received contralateral THA afterwards (second surgery) but only 1 patient had CoM bearing on the other side. In the GROUP B, 7 patients underwent second surgery on the contralateral hip, but only 4 ones received CoM bearing on the other side. The mean postoperative GROUP A follow-up was 97 months (73–125 months). The mean age at time of follow-up was 71.97 years (49–82 years) and the average body mass index (BMI) was 27.91 kg/m2 (21.1–51.4 kg/m2). In the GROUP B, the mean postoperative follow-up was 131.6 months (113–148 months). The mean age at follow-up was 64 years (46–78 years) and the mean BMI was 27.78 kg/m2 (22.7–33.9 kg/m2). Totally, 49 CoM bearings were analysed at a mean follow-up of 114.3 months. (Table 1).

**Table 1:** Demographics of the three groups analysed. Cr: chromium; Co: cobalt

	GROUP A (n:19)	GROUP B (n:25)	CONTROLS (n:20)
Age, years (mean, SD)	*71.97, 9.39*	*64, 9.51*	*70.8, 9.45*
Sex, male (n, %)	*10, 53%*	*17, 68%*	*8, 40%*
Osteoarthritis, primary (n, %)	*18, 95%*	*17, 68%*	–
Side, right (n, %)	*9, 47%*	*12, 48%*	–
BMI, kg/m^2^ (mean, SD)	*27.91, 6.51*	*27.78, 3.24*	*–*
Weight, kg (mean, SD)	*76.5, 18.25*	*83, 13.58*	*–*
Follow-Up, months (mean, SD)	*97, 16.15*	*131.6, 10.05*	–
Cr, μg/l (mean, SD)	*2.16, 2.98*	*3.16, 4.00*	*3.22, 3.25*
Co, μg/l (mean, SD)	*0.85, 1.55*	*2.59, 6.18*	*0.39, 1.72*

Patients enrolled in both groups were evaluated, preoperatively and at last follow-up, clinically in terms of Harris Hip Score (HHS), Visual Analogue Scale (VAS), Western Ontario and McMaster Universities Osteoarthritis Index (WOMAC), 12-item Short Form Health Survey (SF-12P) (physical) and SF-12 M (mental). All the patients underwent to anteroposterior (AP) pelvis radiographs, taken before surgery and after the procedure and at the last follow-up, and the exams were analysed and measured by 3 of the authors in a blinded fashion and in a random order using AXIOVISION 4.8.2 software (Carl Zeiss Microimaging GmbH), by a validated and previous descripted methodology [14]. The measurements allow to define how much the correct joint geometry was reproduced after surgery: off-set, cervical-diaphyseal angle and leg length discrepancy (> 1 cm was considered significant). We also evaluated, on the last radiograph, the presence of subsidence cup inclination and anteversion. Stress-shielding, spot-welds, cortical hypertrophy and femoral osteolysis were graded at final follow-up according to the classification of Engh et al. Short stem radiological outcome was assessed according to a modified Gruen zoning system, eliminating zones 3 and 5 [15]. We also evaluated metal-back osseointegration according to Hodgkinson’s classification. Periprosthetic heterotopic ossifications were evaluated by Brooker’s classification (from 1 to 4).

Analysis of serum Cr and Co was performed at last follow up by Inductively Coupled Plasma mass spectrometry (ICP-MS) (Agilent Technologies, Palo Alto, CA) fitted with an ASX510 autosampler (CETAC, Omaha, NE, USA). Analysis was carried out by switching between two acquisition modes: normal and cool plasma conditions for quantification of Co as 52Co and Cr as Cr, respectively. A detailed description of the operating conditions is available elsewhere. Accuracy was verified by using a certified reference material (ClinChek Serum Control, lyophil., for Trace Elements, Level I, Recipe Chemicals + Instruments GmbH, München, Germany). Venous blood samples were collected using polypropylene tubes and 21-gauge stainless steel needles. All collection tubes and containers were determined to be free of trace metal, and care was taken to prevent metal contamination, using gloves without powder and isopropyl alcohol for disinfection [16]. An accurate interview with the patients was conducted with the aim of exclude the presence of other implanted medical devices containing Cr-Co and occupational exposure to metals. The possible intake of supplements containing Cr was also investigated. According to current knowledge, levels < 2 μg/l seem to be non-critical, levels between 2 and 7 μg/l are considered borderline with unknown biological consequences and levels > 7 μg/l indicate a problem which should be further diagnosed and eventually treated.

The venous blood sample values obtained from Group A and B were compared with those of a control group (GROUP C) made of 21 healthy people, homogenous for demographic characteristics, enrolled after an accurate anamnestic interview.

A value of 7 μg/l was considered the cut-off for risk of adverse reaction to metal debris (ARMD), as indicated by MHRA [11].

Statistical analysis was performed using SPSS (IBM Statistics). Paired t-test or Wilcoxon signed rank test was applied to assess changes after the surgical procedure. Intra-class correlation coefficients were used as a measure of concordance in radiographic evaluations between surgeons. Taking into account baseline differences among groups, the propensity score (PS) was computed according to logistic regression analysis. Thus, chi-square and t-test were used to compare groups. Cr and Co ion levels in our sample followed a log-normal distribution and thus log-transformation enabled is to obtain a good fit to Gaussian. A confidence level of 95% was selected and a *p*-value< 0.05 chosen as significance threshold.

The study was approved by the local ethical committee and each patient enrolled expressed written informed consent to participate.

## Results

All the implanted stems were well positioned and osseointegrated at a mean follow-up of 114 months (min:73; max: 148). There were no major postoperative complications in both groups, such as vascular or nerve injuries or infections.

In both groups, there was a marked improvement in all the parameters compared to the preoperative conditions. Pain was significantly reduced in both groups, (VAS of GROUP A vs. GROUP B) from 66 to 4.5 and from 72.4 to 5.5 (t: 17.23; *p* < 0.001 for GROUP A; t: 19.32; *p* < 0.001 for GROUP B). Marked improvement was also documented for HHS and WOMAC in both groups: HHS of GROUP A increased from 51.6 to 95.3, and in GROUP B from 48 to 98 (t: − 11.57; *p* < 0.001 for GROUP A; t: − 28.87; p < 0.001 for GROUP B); WOMAC of GROUP A increased from 48 to 89.4, and in GROUP B from 45.6 to 93.2 (t: − 8.05; p < 0.001 for GROUP A; t: − 8.65; p < 0.001 for GROUP B).

**Fig. 1:**
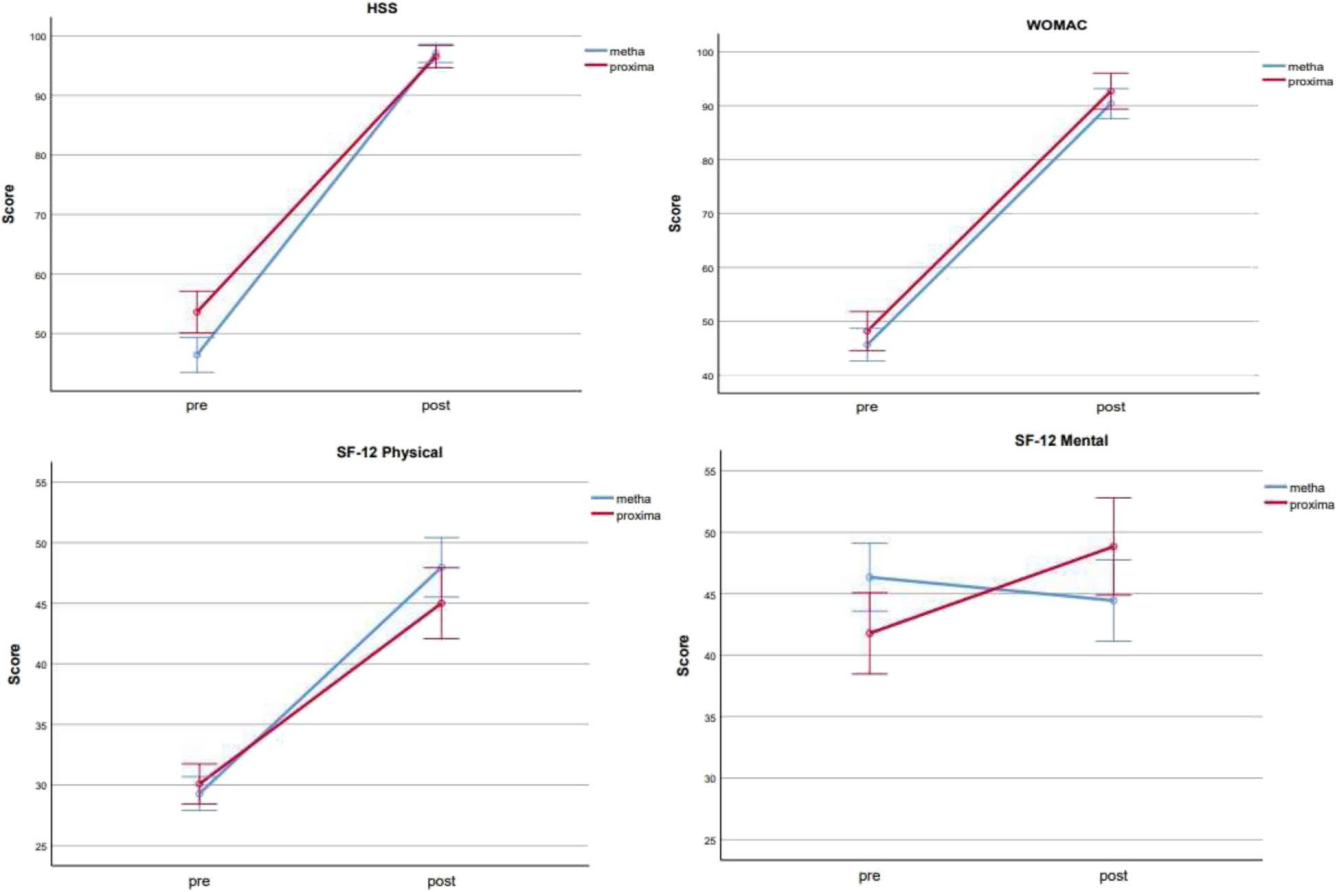
Profile plots showing the clear clinical improvement from the pre-operative time to the follow-up time in the GROUP A and B

There was also an improvement of SF-12 P and SF-12 M, but only GROUP B achieved statistical significance (SF-12 P of GROUP A from 29.9 to 43.6; t: − 1.33; p 0.188; SF-12 M of GROUP A from 43 to 43.5; t: 0.18; p 0.052. SF-12 P of GROUP B from 29.1 to 49.1; t: − 4.89; *p* < 0.001; SF-12 M of GROUP B from 45.1 to 49; t: − 5.36; p < 0.001). (see Fig. 1).

Performing the three groups’ comparisons, it shows that the patients in the GROUP A are older (9.5 years more than the patients in the GROUP B, p < 0.001), while the age difference is significantly smaller with the control group and is not statistically significant (2.7 years more than controls). In conclusion, Group B is younger on average than both other groups.

The follow-up of GROUP B is 40 months longer than that of GROUP A and for this reason the follow-up time will be considered in the subsequent analyses to take it into account.

A greater presence of males was also observed in group B (68%) compared to controls (40%) and group A (53%). However, this difference was not statistically significant (*p* = 0.145).

Because the 2 groups (GROUP A and B) did not match perfectly in terms of age and follow-ups, it was decided to compute the PS taking into account the baseline differences.

After adjusting the analysis considering the differences between the two groups (especially the age of the patients in GROUP A), the two prostheses showed very similar clinical results at last follow-up.

SF-12 P and SF-12 M seem to be statistically better in the GROUP B (SF-12 P: t − 4.39; p 0.0003; SF-12 M: t − 4.59; p 0.0001). No differences were found for HHS (t: − 1.26; p: 0.215), VAS (t: − 0.30; p: 0.769) and WOMAC (t: 1.82; p: 0.074).

It was then analysed whether the improvement in clinical scales was statistically significantly correlated to the implanted prosthesis model or to co-variables such as BMI, age, time of follow-up. No significant interactions were detected, indicating that the improvement seen after implanting Proxima is no different from the improvement seen after implanting Metha. Therefore, it can be said that clinical improvement is independent from the type of the short stem implanted. Not even the other variables have an effect, if not marginally the age on the quality-of-life scales, as the improvement is greater in younger patients (GROUP B). In fact, the mean SF-12 P of GROUP B was 49.1 (vs. 43.6 of GROUP A) and the mean SF-12 M of GROUP B was 49 (vs. 43.5 of GROUP A).

Radiographic evaluations showed high concordance correlation between the 3 blinded surgeons (intraclass correlation coefficient [ICC] consistently > 0.80), showing a good ability to restore proper articular geometry in both groups (off-set of GROUP A: 41.84 vs. 38.48; *p* = 0.0722; cervical-diaphyseal angle of GROUP A: 129.62° vs. 126.09°; *p* = 0.0422; off-set of GROUP B: 46 vs. 39.8; *p* = 0.763; cervical-diaphyseal angle of GROUP B: 133 vs. 127; *p* = 0.937).

Leg-length discrepancy (> 5 mm) was highlighted in 6 patients, without any complaint, in GROUP A and in 15 patients in GROUP B. Cortical hypertrophy was present in 1 patient in zone 4 and 1 patient in zone 6 in GROUP A and in 4 patients in zone 2 and 1 patients in zone 6 in GROUP B. Implants showed stress shielding in zones 1 and 7 (GROUP A: 62%; GROUP B: 75%) and spot-welds in zones 2 and 6 (GROUP A:75%; GROUP B: 99%) probably due to the femoral stem design. Calcar atrophy was evident in 5 patients in the GROUP A and in 8 patients of GROUP B. 8 femoral stems were undersized in the GROUP A and 2 stems in the GROUP B, but this did not influence the survival of the implants.

Femoral osteolysis was only documented in 2 patients in zone 1 in GROUP A.

No radiolucent lines or severe osteolysis around the surface of the femur and the cup (rarely in not more than 1 or 2 Charnley-De Lee zones) were observed in both groups. No progressive axial subsidence > 5 mm was observed in both groups.

10 patients were affected by heterotopic ossification (Brooker 1 in 5 cases; Brooker 3 in 5 cases) in the GROUP A and 11 patients in the GROUP B (Brooker 1 in 7 cases; Brooker 2 in 3 cases and Brooker 3 in 1 case). The cup was well positioned in both groups (inclination mean: GROUP A 47.91° min 40.2–max 60.1 vs. GROUP B: 52.14° min 42.1-max 58.2 - anteversion mean: GROUP A 13.98° min 4.42–max 22.33° vs. GROUP B: 6.49 min 0.82-max 17.08).

Generic risk factors that could induce an increase in Cr-Co serum levels were investigated. No patients had risk factors, except for the CoM bearing, in the GROUP A. 2 patients of the GROUP B were taking a dietary supplement containing Cr when venous blood sample was collected. Renal function was not affected during the study; creatinine levels were within the normal range for sex and age. Serum levels of Cr and Co metal ions were detectable in all patients of both groups.

Mean Cr and Co ion levels in the GROUP A were 2.16 μg/l (0.18–13.1 μg/l; standard deviation [SD] 2.98) and 0.85 μg/l (0.15–6.96; SD 1.55). Cr and Co levels in the GROUP B were 3.16 μg/l (1.26–21.27; SD 4.00) and 2.59 μg/l (0.42–30.6; SD 6.18). Analysing in detail GROUP A and GROUP B vs. controls, the Cr levels were not different in the three groups, while the Co levels appeared significantly higher in the GROUP B than both controls and GROUP A.

However, such estimations are affected by the asymmetry of the distributions. Thus, after log-transformation and back-transformation to the original scale, more accurate estimations of Cr and Co were obtained. After covariating by age, BMI and time of follow-up, multivariate assessment of difference between the two stems in terms of ion levels of both Cr and Co indicated a significant effect (Hotelling T 0.198; df = 2, 36; p 0.039). Looking at each metal, Cr was lower after Proxima implant (p 0.012) while the difference, although in the same direction, did not reach the significance threshold in terms of Co ion levels (p 0.062). Comparing Cr and Co ion values in the three groups (including controls), no significant difference emerged between them (p 0.181), demonstrating that the CoM bearing implantation did not involve an increased risk of metallosis.

**Fig. 2:**
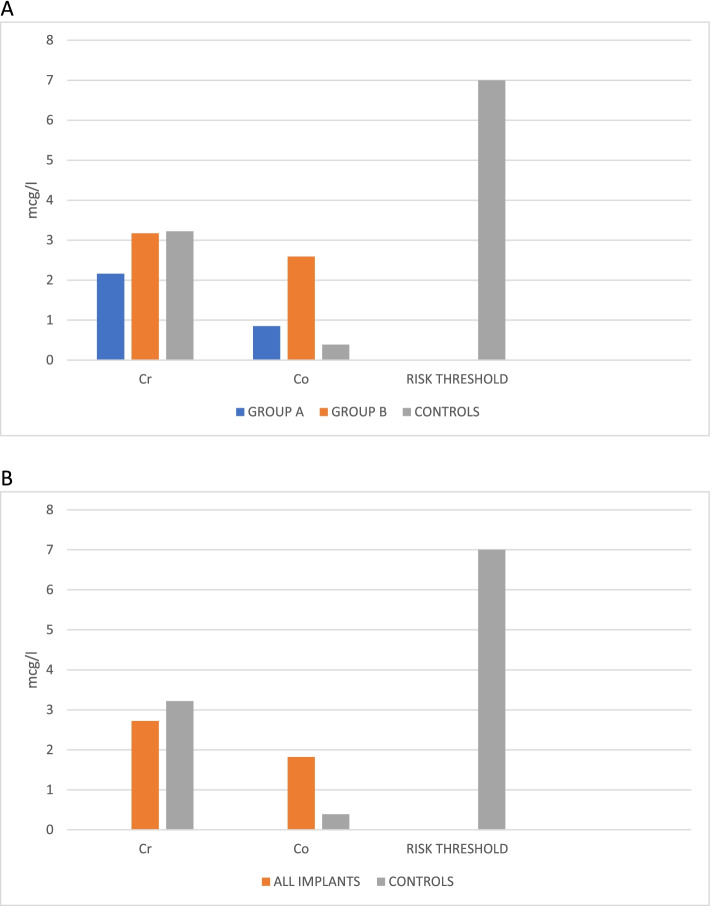
Histograms showing the Cr and Co ions level in the three groups **(A)** and in all patients analysed versus controls **(B)**. Co levels appeared significantly higher in the GROUP B than both controls and GROUP A. All the mean values remained below the risk threshold of 7 μg/l

We then compared all the implanted prostheses (regardless of the type of femoral stem used) with the controls, obtaining a statistically significant difference for blood ion elevation in the prosthesis group only for Co (p 0.018). (see Fig. 2).

In any case the mean values remained below the risk threshold of 7 μg/l.

**Fig. 3:**
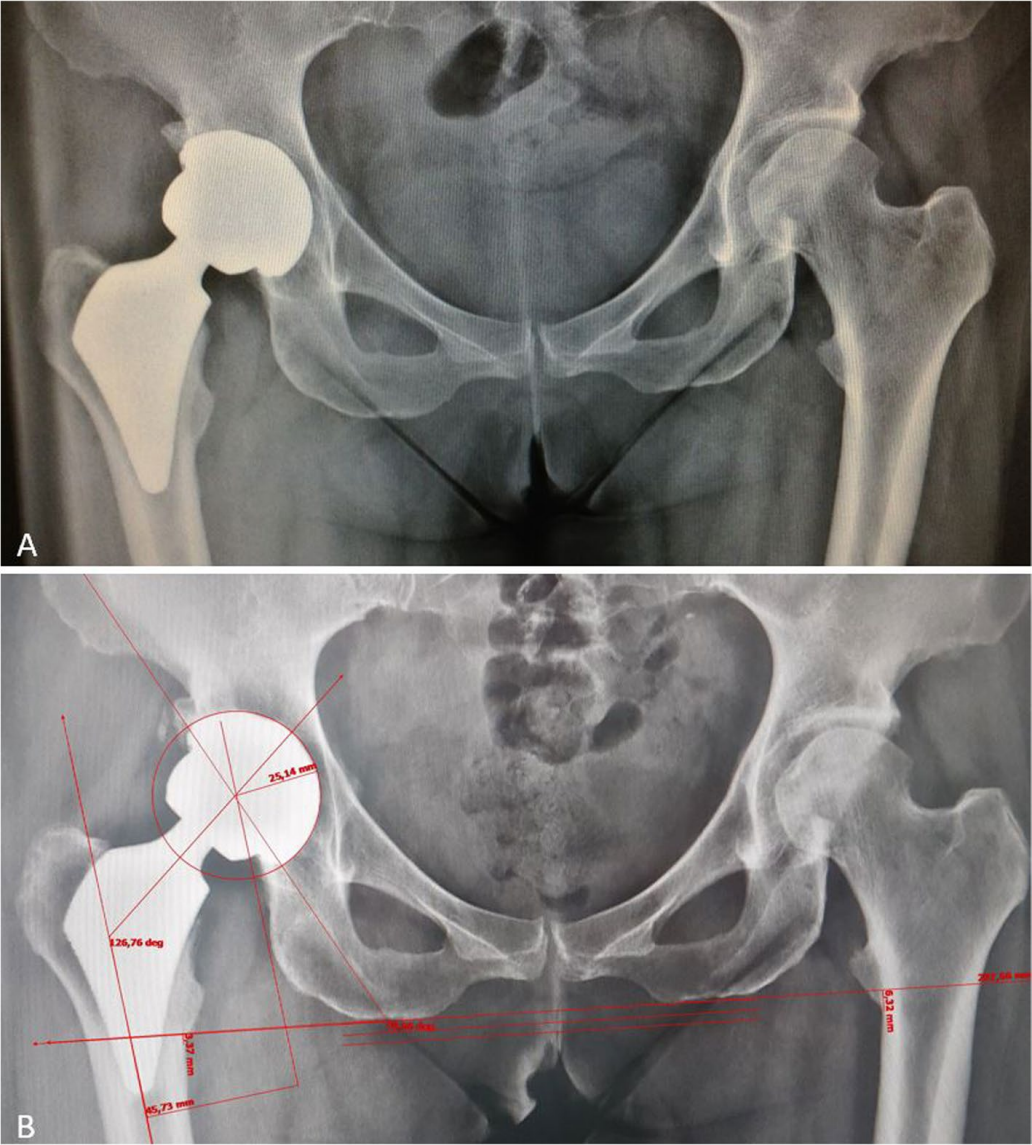
X-ray of a GROUP A (Proxima stem on CoM) patient. **A:** 3 months after surgery: excessive inclination of the cup is evident (A). **B:** At 77 months of follow up the patient was asymptomatic, but high levels of chromium and cobalt were recorded. The X-ray showed cup inclination of 59,6° in a well osseointegrated prosthesis and in absence of any sign of bone resorption, pseudotumor or failure

**Fig. 4:**
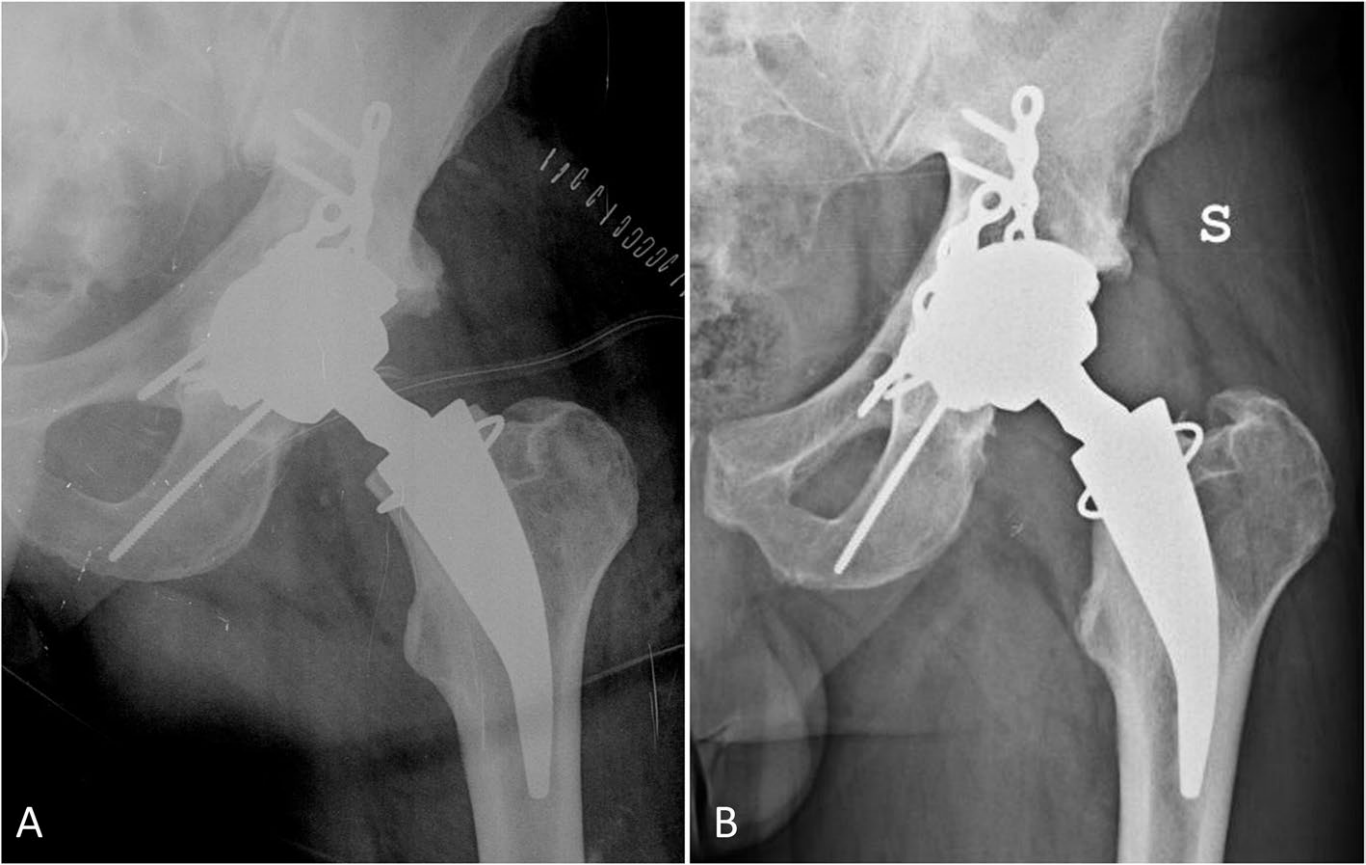
Group B X-ray (Metha stem on CoM) in a patient suffering from secondary osteoarthritis post acetabular fracture. **A:** post-operative X-ray showing a well-positioned implant; **B:** last-follow-up X-ray (142 months) in an asymptomatic patient but with very high levels of chromium and cobalt, showing only calcar neck resorption in absence of any sign of pseudotumor or failure. The implant is well osseointegrated

In 3 cases (6.8%), 1 Proxima and 2 Metha, metal ions were detected to be significantly above the safe ranges without signs or symptoms of local or systemic metal toxicity. Excessive inclination of the cup (59.66 °) was recorded in 1 female patient (Fw-Up:77 months) affected by primary osteoarthritis (OA), and this correlated with an increase in blood Cr and Co levels (Cr: 13.1 μg/l; Co:6.96 μg/l). (see Fig. 3) In the other two cases, one secondary to DDH (Fw-Up:146 months; Cr:6,36 μg/l; Co:8.22 μg/l), and the other consequent to acetabular fracture (Fw-Up:142 months; Cr:21,27 μg/l; Co:30.59 μg/l), the implants were well positioned. (see Fig. 4) All the implants showed to be well osseointegrated with minor signs of local osteolysis or calcar atrophy. Even clinical results were good to excellent (HHS:84–100; VAS:0; SF 12 P: 46,6–56; SF 12 M:19–61; Womac: 92–100). Actually no one of them required revision surgery even if proposed by the surgeons.

Resuming, the two cohorts (Group A and Group B), 44 patients, 49 CoM bearings, at a mean follow up of 114 months showed osseointegration in 100% of cases, with a significant increase in clinical scores (HHS:96.8; SF-12 P:46.7; SF-12 M:46.6; WOMAC:91.5) and decrease in pain (VAS:5.07).

The 44 CoM enrolled patients’ ions levels did not differ significantly from the control group and no revision was required during the follow-up period. On the other hand, considering the other missed patients (*N* = 80), contacted by telephone, 4 cases of revision and 2 suspected pseudotumor were referred in the Group B. Counting all the 124 CoM patients, for what was possible to document, it means a revision rate of 3.2% and a failure rate (revision plus pseudotumor) of 4.6%.

## Discussion

Research in hip prosthetic surgery has the main target to improve the materials in order to ensure the efficacy and safety of the implants. Hard-on-hard bearings have advantages and disadvantages. In particular, CoC is currently the bearing with the best clinical results, guaranteeing low wear rates, biologically inert debris and long implant survival. On the other hand, the risk of fracture (greatly decreased over the years) and the emission of acoustic noises are possible complications [17]. At the same time, MoM bearing has long been quite dismissed as, especially in the case of cup malpositioning, it involves the release of Cr and Co ions into the blood with the risk of local and systemic metal toxicity [18]. In order to optimize the benefits and reduce the disadvantages, the CoM bearing was introduced. In consideration of MoM and CoC potential complications, CoM was thought to represent an optimized combination of bearing surfaces without the added risk of ceramic fracture, acoustic noise, or the damaging effects of local and systemic elevated metal ion levels generated at the bearing surface. This last point has long been the subject of debate, as metal ions levels are not normal following CoM arthroplasty, but certainly lower than the levels recorded with MoM bearing. In addition, the metal ions elevation in the blood was within threshold, except in cases where the prosthetic components, in particular the metal-back, had been mal-positioned [13].

Furthermore, the CoM bearing has potential advantages over other bearing surfaces: the metal liner can be thin allowing for 36-mm ball heads. In fact, it is known that the use of large diameter femoral heads improves the stability of the implant, increasing the “Jump Distance”. An additional benefit of this bearing surface is the ceramic head interface with the femoral stem. Studies have demonstrated lower corrosion at the morse taper when ceramic heads were used [19]. The objective of this study was to evaluate the reliability and safety at long term follow-up of the CoM bearing by comparing two different prosthetic implants with the same joint bearing and therefore these with a healthy control group made up of patients who do not undergo hip arthroplasty and with similar demographic and anamnestic characteristics.

This study involved 44 patients with an average follow-up of over 9 years. In the literature, it is possible to find several papers on the subject, but few of them have a such long term follow up [20, 21]. We were able to appreciate how all the implants were optimally osseointegrated and no patient needed revision. This finding is important when we think on how many surgical revisions have been performed for metal ion toxicity in MoM bearing bearers. Similarly, it was possible to observe how the increase in blood metal ions, although present, was limited and below the safety threshold. This did not occur in only three asymptomatic patients (6.8%) (1 in the GROUP A and 2 in the GROUP B) with ions levels well above the safe threshold. In the first case these finding had been correlated to cup malpositioning while in the two other cases, it was not directly correlated to surgical technical errors and therefore was unexplainable. Moreover, considering all the CoM (*N* = 124) we found a 3.2% revision rate and a 4.8% failure rate (revision plus pseudotumor). These results are similar to other studies and approaches the revision rates of other bearing combination for total hip arthroplasty, 3.8–6.7% [19].

Mehta et al. reported a revision rate of 3 out of 66 CoM patients (4.5%) and metal ion levels above MHRA thresholds on 6.06% of cases at a mean follow-up of 9 years [20].

Similarly, the average values ​​of Cr and Co metal ions reported in our study are similar to those published in other papers. In 2017, Schouten published a comparison study between CoM and MoM in 83 patients, demonstrating significantly lower values ​​of blood metal ions in the first group, good clinical results and long implant survival [21].

In 2016, Cadossi et al. reported that CoM bearers had higher levels of Cr and Co in their blood compared to the pre-operative time, but still significantly lower than those with MoM at a medium follow-up, with excellent clinical results. Patients were also asymptomatic for local or systemic toxicity due to Cr and Co metal ions [22].

Yi et al., analysing 85 prosthetic hips in 74 patients, concluded that the level of metal blood ions had increased compared to the general population, but that this did not necessarily translate into toxicity in the medium term (50 months) [23].

In 2015, Hill et al. published a case-series of 287 CoM THAs at a follow-up of 34 months, with a survival rate of 97% [10]. (Table 2).

**Table 2:** Characteristics of included studies

	***N. patients***	***Follow-Up (months)***	***Revision Rate (%)***
Metha et al.	66	108	4.5%
Schouten et al.	83	60	5%
Cadossi et al.	20	36	0%
Yi et al.	74	50	0%
Han et al.	19	97	0%
Saracco et al.	36	104.4	0%
Higgins et al.	121	42	30.5%

Furthermore, these evidences on CoM bearing do not seem to be correlated to ethnical differences [24].

On the contrary, on 2019, Higgins reported a dramatic incidence of 30,5% of revisions for ALTR, (7 on 36 CoM implants) at 8.7 years even in presence of metal ions well below the safety threshold [25]. In this case it is to consider that this study was a premarket one, and probably failure was due to hardware technical problems or malposition when used on hard bearings, and the MoM bearing was object of recall by the “System for Australian Recall Actions” (RC-2015-RN-00100-1).

A case report was published in 2017 on a patient with CoM who had suffered from pseudotumor revised 7 years after surgery [26]. Malpositioning of the metal-back (as in our case of group A) strongly affected the reliability of the C-M bearing. In particular, excessive anteversion of the metal-back increases wear and therefore the release of Cr and Co metal ions. Moreover, the literature reports several cases and series which show this close correlation between malpositioning, toxicity and failure [27, 28].

Hart et al. has published an interesting study on this subject: he evaluated the correlation between cup inclination and anteversion and the incidence of ion release and implant failure in MoM bearings using computerized tomography (CT) scan [29]. He showed that patients with metal toxicity and MoM implant failure had cup malpositioning.

Surprisingly, in our study, the healthy control group (Group C), demographically similar and resident in the same region of the studied groups (Group A and B), showed metal ions levels statistically not different from the CoM cases, apparently confirming that CoM bearing implantation did not involve an increased risk of metallosis.

However, comparing the blood Cr-Co levels reported in our study to that found in the general Italian population (published elsewhere) a metal ion rise is evident, but this increase remained well below the safety threshold [13, 30].

In our study it is interesting to note that Co ions level was higher in a non-statistically significant way in patients with Metha femoral stem (GROUP B), although it is not clear why this happens.

Finally, the comparison between the groups shows that the implantation of the CoM bearing does not involve a statistically significant risk of complications, such as local and systemic toxicity from metal ions.

Comparing clinically the two stems, no differences were seen except for SF12 that was better in the Metha group, and was related to the younger age, independently of BMI and time of follow up.

Calcar atrophy and stress shielding were found relatively frequent in both groups. We believe that this was due to the design of the prosthetic stems and to modification of the load lines. In addition, we detected an excellent fixation of the implants and optimal osseointegration confirmed by spot welds in the totality of the cases.

These results confirmed how short stems works well even at long term follow up, as recently described in the 2020 Australian Arthroplasty Register, that report a cumulative revision rate at 15 years of 6.35%, better of 7.8% of other traditional stems, probably because of optimal load distribution across the metaphyseal region favouring proper system integration [14, 19, 31].

Consequently, it is reasonable to deduce that short stems work well also with hard bearings, as CoM coupling.

Our study has some limitations: the retrospective and not blinded design; the lack of repeated clinical and laboratory evaluations over time, the absence of a trend in venous blood Cr-Co levels; the GROUP A and B patients characteristics are not perfectly homogeneous for age and follow up. Furthermore, the study is a multicentric one, based on 3 groups (2 cases and 1 controls group) with two different designs of short femoral stem and CoM bearing studied at long term follow up and compared to a healthy control group. Beyond this, our mean follow-up of 9.5 years is long enough to demonstrate that implants with CoM bearing, when well implanted and in respect of the articular geometry, are safe and effective.

## Conclusions

The results demonstrate that the CoM coupling is a safe and reliable bearing at long term follow up in association to short stems arthroplasty when the implant is correctly positioned. Patients with CoM bearing must be suggested to perform annually Cr and Co metal ions blood levels evaluation even if asymptomatic, as they carry a device that can reasonably be source of release of local and systemic ions. In case of consistent increased values, strictly observation and revision should be considered. Future studies at longer follow up will report the final behaviour of CoM coupling.

## Data Availability

The datasets used during the current study are available on request from the corresponding author.

## Abbreviations

THA: Total hip arthroplasty
CoC: Ceramic-on-ceramic
CoM: Ceramic-on-metal
MoM: Metal-on-metal
Cr: Chromium
Co: Cobalt
ALTR: Adverse local tissue reactions
MHRA: Medicines and Healthcare Products Regulatory Agency
OA: Osteoarthritis
AVN: Avascular necrosis
DDH: Developmental dysplasia of the hip
BMI: Body mass index
HHS: Harris Hip Score
VAS: Visual Analogue Scale
WOMAC: Western Ontario and McMaster Universities Osteoarthritis Index
SF-12P: 12-item Short Form Health Survey Physical
SF-12 M: 12-item Short Form Health Survey Mental
AP: Anteroposterior
ICP-MS: Inductively Coupled Plasma mass spectrometry
ARMD: Adverse reaction to metal debris
PS: Propensity score
ICC: Intraclass correlation coefficient
CT: Computer tomography
